# The IGF-1 Signaling Pathway in Viral Infections

**DOI:** 10.3390/v13081488

**Published:** 2021-07-29

**Authors:** Agata Józefiak, Magdalena Larska, Małgorzata Pomorska-Mól, Jakub J. Ruszkowski

**Affiliations:** 1 Department of Preclinical Sciences and Infectious Diseases, Poznan University of Life Sciences, Wołyńska 35, 60-637 Poznań, Poland; malgorzata.pomorska@up.poznan.pl; 2 Department of Virology, National Veterinary Research Institute, Al. Partyzantów 57, 24-100 Puławy, Poland; maglar7@wp.pl; 3 Department of Animal Anatomy, Poznan University of Life Sciences, Wojska Polskiego 71C, 60-625 Poznań, Poland; jakub.ruszkowski@up.poznan.pl

**Keywords:** IGF-1, IGF-1R, oncogenic viruses, signal transduction, IGF-1 signaling, viral infection

## Abstract

Insulin-like growth factor-1 (IGF-1) and the IGF-1 receptor (IGF-1R) belong to the insulin-like growth factor family, and IGF-1 activates intracellular signaling pathways by binding specifically to IGF-1R. The interaction between IGF-1 and IGF-1R transmits a signal through a number of intracellular substrates, including the insulin receptor substrate (IRS) and the Src homology collagen (Shc) proteins, which activate two major intracellular signaling pathways: the phosphatidylinositol 3-kinase (PI3K)/AKT and mitogen-activated protein kinase (MAPK) pathways, specifically the extracellular signal-regulated kinase (ERK) pathways. The PI3K/AKT kinase pathway regulates a variety of cellular processes, including cell proliferation and apoptosis. IGF1/IGF-1R signaling also promotes cell differentiation and proliferation via the Ras/MAPK pathway. Moreover, upon IGF-1R activation of the IRS and Shc adaptor proteins, Shc stimulates Raf through the GTPase Ras to activate the MAPKs ERK1 and ERK2, phosphorylate and several other proteins, and to stimulate cell proliferation. The IGF-1 signaling pathway is required for certain viral effects in oncogenic progression and may be induced as an effect of viral infection. The mechanisms of IGF signaling in animal viral infections need to be clarified, mainly because they are involved in multifactorial signaling pathways. The aim of this review is to summarize the current data obtained from virological studies and to increase our understanding of the complex role of the IGF-1 signaling axis in animal virus infections.

## 1. Introduction

Insulin-like growth factor-1 (IGF-1) and IGF-1 receptor (IGF-1R) belong to the insulin-like growth factor family, which includes insulin, insulin-like growth factor-2 (IGF-2), their receptors, six IGF-binding proteins (IGFBPs) and 10 IGF-binding protein-related proteins (IGFBP-rPs) [1]. IGF-1 is a secretory protein with a molecular weight of 7.6 kDa consisting of a single peptide chain with 70 amino acids. The liver is the major source of IGF-1 found in blood. In addition to the circulating form produced by the liver, IGF-1 is also produced locally in tissues and exhibits autocrine/paracrine activities on cells. IGF-1 activates intracellular signaling pathways by binding with high affinity to specific IGF-1 receptor isoforms and with lower affinity to a noncognate receptor, e.g., insulin (INSR, insulin receptor). The receptors for insulin, such as growth factor 1 and insulin, are closely related to integrated membrane glycoproteins. IGF-1R is a type 1 transmembrane receptor tyrosine kinase (RTK) that shares ~70% homology with INSR; they both play crucial roles in regulating cell cycle progression, proliferation and apoptosis. Both receptors can also form hybrid receptors (HRs). HRs have a high affinity for IGF-1 and a lower affinity for IGF-2. IGF-1R can also heterodimerize with EGFR [2].

**Figure 1:**
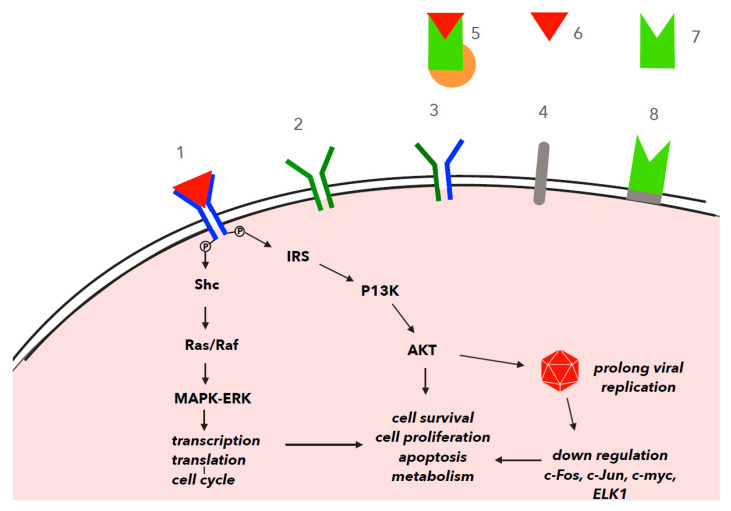
IGF-1 signal transduction via IGF-1 receptor (IGF-1R) in viral infections. The phosphorylated tyrosine residues of IGF-1R act as docking stations for substrates, such as insulin receptor substrate (IRS) and Shc adaptor proteins, and then recruit additional factors to activate two major cascades, the phosphatidyl inositol 3-kinase (PI3K) and mitogen-activated protein kinase (MAPK) pathways. Ras/Raf participates in the activation of the MAPKs ERK1 and ERK2, which phosphorylate and activate several proteins and stimulate cell proliferation. ERK activates a wide variety of substrates that regulate transcription and translation, controlling the cell cycle in some viral infections. Viral activation of the PI3K/AKT signaling pathway slows apoptosis and prolongs viral replication. Some of the important proteins in this pathway are transcription factors such as c-Fos, c-Jun, c-myc and Elk1.1. IGF-1R+IGF-1, 2. IGF-2R, 3. hybrids: IGF-1R/insulin receptor, 4. IR (Insulin receptor), 5. IGF-1+IGFBP+ALS, 6. IGF-1, 7. IGFBP, 8. IGFBP-R+IGFBP.

Generally, IGF-1R signaling is involved in regulating cell growth, whereas INSR signaling regulates carbohydrate metabolism. The interaction between IGF-1 and IGF-1R results in the trans-autophosphorylation of a portion of these receptors, and subsequently, the resulting signals are transmitted through a number of intracellular substrates, including the insulin receptor substrate (IRS) and the Src homology collagen (Shc) proteins, which activate two major intracellular signaling pathways: the phosphatidylinositol 3-kinase (PI3K)/AKT and the mitogen-activated protein kinase (MAPK) pathways, specifically the extracellular signal-regulated kinase (ERK) pathways. The serine/threonine kinase ERK activates a wide variety of substrates that regulate transcription and translation, controlling the cell cycle. Activated PI3K leads to increased phosphatidylinositol 3,4,5-triphosphate (PIP3) levels, resulting in the activation of the AKT/PKB protein through phosphorylation. The PI3K/AKT kinase pathway regulates a variety of cellular processes, including cell proliferation, RNA processing, protein translocation, autophagy and apoptosis [3]. It also plays an important role in the induction of antiviral responses. Many viruses benefit from activating, not suppressing, the PI3K/AKT signaling pathway. Viral activation of the PI3K/AKT signaling pathway slows apoptosis and prolongs viral replication. On the other hand, PI3K/AKT activity is associated with upregulating the interferon response [4]. Generally, when apoptosis is blocked by a virus, the PI3K/AKT signaling pathway induces the expression of interferon-responsive genes. IGF1/IGF-1R signaling also promotes cell differentiation and proliferation via the Ras/MAPK pathway. When IGF-1R activates the IRS and Shc adaptor proteins, Shc stimulates Raf through the GTPase Ras. Raf participates in the activation of the MAPKs ERK1 and ERK2, which phosphorylate and activate several proteins and stimulate cell proliferation. Phosphorylation is a key process in signal transduction that can activate or inhibit downstream signaling proteins [5]. The Ras/Raf/MEK/ERK pathway is required for some viral infections [6]. Some of the important proteins in this pathway are transcription factors such as c-Fos, c-Jun, c-myc and Elk1 [7] (Figure 1).

## 2. IGF-1 and IGF-1R

The IGF-1 gene in the human genome is located on chromosome 12 and extends to 85 kb. The gene comprises six exons separated by introns, producing alternative class 1 and class 2 transcripts. Exons 1 and 2 are differently spliced to exons 1 and 3, producing alternative class 1 and class 2 transcripts. The alternative splicing of exons 5 and 6 gives rise to six IGF-1 precursors: IGF classes 1A and 2A contain exons 3–4 and 6 of the transcript and form the IGF-1 Ea isoform with a C-terminal Ea extension peptide. IGF-1B and IGF-2B contain exons 3–5 (IGF-1Eb) isoform, and the class 1C and 2C isoforms (IGF-1Ec) arise from a splice site within exon 5, which links 49 nucleotides of exon 5 to exon 6 [8]. Mature IGF-1 is encoded by exons 3 and 4.

IGF-1 binds to these IGF receptor isoforms, insulin receptors and hybrid receptors consisting of IGF-1R and INSR. IGF-2 is the only ligand for IGF-2R [9]. After IGF-1 binding, the beta subunits of IGF-1R undergo autophosphorylation through their respective tyrosine kinase domain. The phosphorylated tyrosine residues of IGF-1R act as docking stations for substrates, such as insulin receptor substrate (IRS) and Shc adaptor proteins, and then recruit additional factors to activate two major cascades, the phosphatidyl inositol 3-kinase (PI3K) and mitogen-activated protein kinase (MAPK) pathways (Figure 1).

IGF-1R undergoes both caveolin- and clathrin-mediated endocytosis. Upon internalization, IGF-1R is transported to endosomes, from which it is transported back to the cell surface for recycling or to lysosomes for degradation [10]. IGF-1R has also been shown to be translocated to the cell nucleus in humans with novel types of normal and cancerous cells. IGF-1R is localized to the perinuclear and nucleolar areas of the nucleus, which is regulated by the SUMOylation of IGF-1R at three evolutionarily conserved lysine residues (K1025, K1100, and K1120) in each beta subunit [11].

Nuclear IGF-1R is phosphorylated in response to ligand binding and undergoes IGF-induced interactions with chromatin, suggesting its participation in the regulation of transcription [2,3,12,13]. For example, nuclear IGF-1R binds the transcription factor LEF-1, a key regulator of the Wnt signaling cascade, and acts as a coactivator of LEF-1/TCF target genes [14]. Wnt and MAP kinase-mediated cell signaling is also involved in the oncogenesis induced by the bovine leukemia virus (BLV) [15].

IGF-1R expression is also regulated by the mRNA level of the host transcription factors and posttranscriptional modifications by miRNAs. It has been shown that a high level of IGF-1R expression is required for maintaining leukemia stem cells (LSCs) [2].

IGF-1R is known to promote tumorigenesis and resistance to cancer therapeutics. IGF-R overexpression is associated with a poor prognosis for patients with one of several tumor types, including non-small cell lung, pancreatic, colorectal, ovarian and head and neck squamous cell carcinomas (HNSCCs) [16]. Dale et al. (2015) indicated that IGF-1R overexpression is associated with low survival, HPV negativity and high tumor T-stage in HNSCC [17]. IGF-1 in the human serum signaling axis is required for cell transformation and promotes cancers. The IGF-1R gene transcription rate depends on a number of stimulatory nuclear proteins and is modulated by negative transcriptional regulators, including p53, p63, p73 and BRC-1 [12,13,14,18,19,20]. IGF-1R in combination with predictive molecular markers could serve as a promising approach in anticancer therapeutics.

## 3. IGFBPs

The serum level of IGF-1 and its bioavailability and bioactivity are controlled and modulated by six IGF-binding proteins (IGFBPs) [21]. In circulation, IGFBP1–5 has the same affinity for IGF-1 and IGF-2, while IGFBP-6 has a binding preference for IGF-2 [1]. Approximately 99% of circulating IGF-1 is bound to IGFBPs, with most bound to IGFBP-3, which is the most abundant IGFBP in human serum. The key function of IGFBPs (especially IGFBP-3 and, to a lesser extent, IFGBP-5) is the formation of ternary complexes with IGF and the acid–labile subunit (ALS), which increases the half-life of unbound IGF-Rs. IGF-1 may be “inactivated” after it binds with IGFBP-3. IGFBP-3 expression has also been shown to suppress the activation of IGF-1R, AKT and EKR signaling significantly. IGFBP-3 can also form a complex with the chaperone protein GRP78, which induces apoptosis by competing with caspase 7 for GRP78 binding [22].

IGFBP-3 can be internalized to the nucleus and interacts with nuclear hormone receptors, such as retinoid X receptor, retinoid acid receptor and vitamin D receptor, which induces the transcription of IGFBP3, IGFBP-4, IGFBP-5 and IGFBP-6 and the inhibition of cell growth [23,24,25,26]. High levels of circulating IGF-1 and low levels of IGFBP-3 are associated with an increased risk of several cancers, including those of the prostate, breast, colon and lung. IGFBPs may act independently of a receptor, inducing mitogenesis and cell migration. IGFBP-2, -3 and -5 contain a nuclear localization signal and may also influence transcription. The transcription factor for IGFBP-3 might be methyl CpG-binding protein 2 (MeCP2). Research performed on tissues with known neurological disorders showed that MeCP2 can bind to IGFBP-3 in the cell nucleus and directly regulate the expression of the IGFBP-3 gene [27].

IGFBP-2 can act independently of IGFs by interacting with cell surface-, intracellular- or nuclear-binding partners. Nuclear IBFBP2 can activate the VEGF expression and regulate angiogenesis [28]. Most IGFBPs may have oncogenic potential. For example, IGFBP-1, -2 and -6 can stimulate cell migration by interacting with alpha 5 integrin and prohibin [29,30]. IGFBP-2 is highly expressed in the serum and tumor tissues of most cancers. IGFBP2/5 can participate in antiapoptotic mechanisms via regulation of ERK/MAPK activation [31]. Some IGFBPs can potentially be predictive and prognostic biomarkers of cancers [23]. A strong association has been observed between gliomas and IGFBP-2 or IGFBP-1 and prostate cancer [29,30].

## 4. IGF-1 in Cell Signaling and Viral Infection

Many viruses not only require host signaling processes for their replication but also actively manipulate host signal transduction [32]. Certain viruses, such as avian leucosis virus (ALV), African swine fever virus (ASFV), enterovirus 71 (EV71), Zaire Ebola virus (ZEBOV) and hepatitis C virus (HCV), utilize the PI3K/AKT signaling pathway during the host cell entry [7,9]. Tax genes, which are key contributors to the oncogenic potential of BLV, are also regulated via Wnt-mediated signaling and MAP kinase signaling. Therefore, inhibition of PI3K reduces infection by all viruses. The PI3K/AKT signaling pathway is also often triggered by viruses, inducing viral splicing, translocation and survival (Figure 1) [32].

For example, AKT induces the inhibitory phosphorylation of proapoptotic molecules such as BAD and caspase cascade reactions to inhibit the phosphorylation of transcription factors such as FOXO1. This specific blockage prevents FOXO1 translocation into the nucleus and thus inhibits the expression of proapoptotic target genes [32]. Viral proteins can also interact with the host cell members of signaling pathways. For example, nonstructural protein 1 (NS1) of influenza A interacts with and activates the PI3K/A pathway via any-apoptotic signaling [33].

The contribution of IGF-1 and IGF-1R to the mechanism of infection caused by the pneumonia virus has been well documented by Griffiths et al. (2020) in research they conducted on the respiratory syncytial virus (RSV) [34]. The authors indicated that IGF-1 binding to IGF-1R is involved in a mechanism of RSV entry into cells involving glycoprotein RSV-F expressed on the virion surface. The fusion of RSV with IGF-1R triggers the activation of protein kinase C zeta, which promotes cell signaling that recruits nucleolin from the nucleus to the plasma membrane [34]. Nucleolin is a coreceptor for RSV and for influenza, parainfluenza (e.g., Peste des petits ruminants virus, PPRV) and calicivirus (e.g., feline calicivirus, FCV) [35]. It is tempting to speculate that other pneumoviruses, including bovine RSV, bind the IGF-1R.

Viral infection can also be regulated by host IGF-binding proteins. This was confirmed in research performed on astrocytes of transgenic mice that express a Borna disease virus (BDV) phosphoprotein (P) [36]. The authors focused on an analysis of the expression IGFBP-3, which binds IGF-1 and regulates the availability of IGF-1 for binding to IGF-1R. Borna disease virus (BDV) is a highly neurotropic virus that belongs to the order *Mononegavirales*. IGF-1 can play a crucial role in the differentiation of neurons and is a neurotrophic factor. Moreover, the abnormal expression of IGFBP-3 was detected in patients with neurological disorders. For example, an increase in IGFBP-3 expression in astrocytes has been shown in the cerebella of transgenic mice expressing the BDV phosphoprotein [36]. In this study, the authors revealed that BDV infection could upregulate the expression of IGFBP-3 and disrupt IGF signaling in infected cells. The authors also suggested that BDV P expression leads to the upregulation of IGFBP-3, possibly through the aberrant expression of methyl CpG-binding protein 2 (MeCP2), a transcriptional repressor [36]. The promoter region of the IGFBP-3 gene contains a MeCP2-binding site, and the expression of the IGFBP-3 gene is directly regulated by MeCP2 [37]. Similar findings with mouse and human brains with Rett syndrome (RTT) have been documented.

The pathogenesis of some viruses is still unclear. Iwakiri et al. (2003) indicated that IGF-1 can serve as an autocrine growth factor in EBV (Epstein-Barr virus) infection in gastric carcinoma [38]. They documented that EBV infection induced the expression of IGF-1 via small components called EBV-encoded small RNAs (EBERs) [38]. The components of the IGF-1 signaling pathway may also involve *miRNAs.* In vitro research performed on NU-GC-3 cells indicated that the transfection of EBV latent genes can induce the production of EBERs and induce IGF-1 expression [39].

Altinds et al. (2018) showed that viruses also carry sequences with significant homology with several human peptide hormones, such as insulin, insulin-like growth factor IGF-1 and IGF-2, fibroblast growth 19 and 21 (FGF-19 and -21), endothelin-1, inhibin, adiponectin and restin [40]. Some of these factors might play roles in the immune response (e.g., restin), while others with the highest homology to insulin and IGF-1 (viral insulin/IGF-1-like peptides, VILPs) might play roles in modulating endocrine systems. Four viruses belonging to the *Iridoviridae* family encode peptides similar to insulin or IGF-1. VILPs show up to 50% homology with human IGF-1, contain all critical cysteine residues in their structures and can form 3D structures similar to insulin or IGF-1. Chemically synthesized VILPs can bind to human IGF-1R and stimulate receptor autophosphorylation, downstream signaling and cellular responses such as proliferation and glucose uptake. VILPs were the first characterized viral hormones. These peptides might affect host pathophysiology by binding to cellular hormone receptors, mimicking the actions of cellular peptides. Altinds et al., using a bioinformatics approach, identified approximately 8000 complete viral genomes with predicted coding sequences with similarity to 16 of 62 tested human peptide hormones, cytokines associated with metabolism and growth factor precursors.

The patients with HCC have been shown to have an increased prevalence of hepatitis B and C virus infection. The impairment of the IGF axis has also been observed in the livers of patients with HCV-related chronic hepatitis. At the hepatocyte level, IGF-1 and nuclear STAT5-p positive scores indicated negative correlations with the fibrosis stage, while the SOCS-3 score showed a positive correlation with the fibrosis stage. In this study, the IGF-1 expression in hepatocytes was reduced with fibrosis progression compared to the control [41]. Circulating IGF-1 levels have been correlated with viral infection and associated with HCC progression. According to Wang and others (2017), the serum level of IGF-1 may be an independent prognostic factor for the progression and survival of HCC patients [42]. The HBV protein XBx plays a role in the process of HBV-associated carcinogenesis. This protein is also critical for the activation of IGF-1R gene expression and the development of HCC.

In both HBV- and HCV-induced HCC, a link with increased IGF-2 expression has been demonstrated. Furthermore, a study showed that IGFB-1 and IGFBP-2 were downregulated in HCC tumor tissue compared with normal liver tissue, whereas IGFBP-4 was upregulated [43].

The gene expression of molecules involved in the PI3K/AKT and Ras/MAPK signaling pathways is controlled by a series of phosphorylation reactions and other modifications (such as methylation, acetylation, ubiquitination and SUMOylation) [44]. In many viral infections, the immune response and activation of certain signaling pathways are required for the viral replication that interferes with host cell signaling (Figure 1) [32,45]. Therefore, a number of viruses have been shown to interfere with the phosphorylation of cellular proteins at all points in signal transduction pathways, from the plasma membrane to the nucleus [5]. For example, within a minute after HIV-1 exposure, more than 200 phosphorylation sites are modified in T cells, which probably alters several cellular processes upon infection and supports viral replication [46]. Through phosphorylation events, viruses can exploit cell signaling pathways for their own replication. The phosphorylation of viral proteins can also regulate the stability, activity and interaction of viral proteins with other proteins [47]. Multiple kinases can phosphorylate the same viral proteins. In addition, some viruses encode their own kinases. Many kinases have characteristic recognition motif substrate sequences that are phosphorylated most efficiently by particular kinases [48].

PI3K activity is significantly increased in laryngeal papilloma, which is induced by the HPV 6/11 virus, leading to the upregulation of EGFR and the subsequent activation of MAPK/ERK. Some RNA viruses require AKT to synthesize viral RNAs (Figure 1) [3].

The PI3k/AKT signaling pathway mediates many cellular and molecular functions through the altered expression of genes that are critical to tumor initiation and progression. It has been shown that human cytomegalovirus (HCMV) infection can activate the PI3 kinase and Akt pathways by major immediate-early proteins (MIEPs), leading to the inhibition of apoptosis [49]. The AKT signaling pathway is also activated by Simian virus 40 large T antigen. The AKT signaling pathway is an important regulator of cell survival (Figure 1). AKT can phosphorylate a number of factors to ensure cell survival, such as Bad, caspase 9, the forkhead family of transcription factors and IkB kinase, which leads to the phosphorylation of IkB and NF-kB nuclear entry [50].

The activation of the PI3K/AKT/mTOR pathway has also been observed during HPV 16/18 infection of human epithelial cells. During HPV carcinogenesis, epithelial cells are immortalized and transformed by the viral oncogenes E6/E7, which are involved in multiple events, including the inhibition of p53 and pRb and the activation of several signaling pathways, especially the PI3K/AKT/mTOR pathway. The PI3K/AKT signaling pathway in HPV-infected cells can also be activated through the mutation of signaling pathway molecules [51].

The main HPV 16 and 18 viral oncoprotein E6 contains a PDZ-binding site that plays a key role in HPV-mediated cell transformation. The PDZ domain-containing viral E6 protein can degrade molecules whose expression is mediated by the PI3K/AKT signaling axis. HPV variants of the E6 oncoprotein can act as adaptor molecules linking a ubiquitin ligase to target proteins that contain PDZ domains [52]. E6 proteins of oncogenic HPV types can also activate the MAPK signaling pathway to promote cell proliferation by upregulating pPI3K expression. HPV16 E6-mediated NHERF-1 degradation correlates with the activation of the PI3K/AKT pathway. HPV 16 E7 oncoproteins also participate in this process by activating cyclin-dependent kinase complexes to promote the accumulation of a phosphorylated form of NHERF-1 that is preferentially targeted by E6 [53,54]. The HPV E7 oncoprotein can directly activate AKT by phosphorylation, leading to the phosphorylation of BAD. This phosphorylation of P13K/AKT pathway components is associated with Notch1 signaling [55]. Moreover, the HPV E7 oncoprotein inhibits the functional cyclin-dependent kinase inhibitors, p21^Cip1^ and p27^Kip1^, by phosphorylating PI3K/AKT signal transduction factors [56].

The activation of AKT induced by HPV E7 expression also plays a crucial role in immune resistance. The oncogenic potential of E6/E7 HPV proteins also depends on IGFBP2. The expression of HPV 16 E6 and E7 oncoproteins causes the reduced expression of IGFBP2 and correlates with the progression of cervical cancer from CIN I to CIN III [16]. Picard et al. suggested that prolonged expression of the E6 and E7 oncoproteins can generate anti-invasive epithelium activity through the depletion of IGFBP2 expression, which in turn leads to signaling through IGFR. IGFBP-2 can increase IGF-1 and -2 signaling and cell invasion. In the presence of IGFBP-2, IGF-1 cannot be released from the cell surface and cannot activate IGF-1R. When IGFBP-2 is lost, KGF activation of ADAM17 leads to the activation of IGF-1R through unprotected IGF and subsequent AKT activation. The activation of AKT by keratinocyte growth factor (KGF) is dependent on IGF-1R and can be modulated by IGFBP-2 and the metalloprotease ADAM1.

In HPV-induced carcinogenesis, the PI3K/AKT mammalian target of the rapamycin (mTOR) signaling cascade plays a very important role through its effect on multiple cellular and molecular events. mTor kinase integrates signals from a variety of cellular signaling pathways. mTor activation has been observed in HPV-related cervical squamous cell carcinomas, such as cervical carcinoma, head and neck squamous cell carcinoma (HNSCC) and oropharyngeal cancers (OPSCCs).

The PDZ motif of the HPV16 E6 oncoprotein plays a key role in HPV-mediated cell transformation. This oncoprotein has the ability to efficiently degrade members of the PDF motif-containing molecules. For example, the interaction of E6 with the PDZ protein-containing NHERF-1 (Na/H exchange regulatory factor 1) promotes its degradation via the proteasome pathway. The phosphorylated form of NHERF-1 is preferentially targeted by E6. NHERF-1 degradation correlates with the activation of the PI3K/AKT signaling pathway [53]. The HPV E7 oncoprotein can also inhibit retinoblastoma protein and stimulate the PI3K/AKT pathway. HPV E6 protein activates several carcinogenic pathways and inhibits the tumor suppressor protein p53 and proteins containing the PDZ domain. The survival pathway activated by E6 includes PI3K/AKT kinases, and Wnt and Notch activation of the PI3K/ATK pathway has been associated with increased cancer cell proliferation, decreased apoptosis and increased cell migration (Figure 1).

The PI3K/AKT signaling pathway mediates many cellular and molecular functions through the altered expression of genes that are critical to tumor initiation and progression. PI3K also modulates different signaling pathways to prevent apoptosis and promote cellular survival and proliferation (Figure 1) [57].

IGF-1R signaling via PI3K-AKT and ERK inhibits the expression of the proapoptotic Bcl-2 family member BAD by maintaining its phosphorylation status [32,58]. The BCL-2 family comprises both antiapoptotic and proapoptotic proteins [57]. The regulation of BCL-2 family proteins is tightly connected with a prosurvival signaling network including NFkB PI3 kinase [59]. Phosphorylation of BAD prevents its heterodimerization with the antiapoptotic Bcl-2 family members Bcl-_xl_ and Bcl-2 [58]. The antiapoptotic function of other proteins, such as BCL-2, BCL_XL,_ BFL-1 and MCl-1, can also be modulated by phosphorylation. The proapoptotic protein BIM-EL is also maintained at low levels through phosphorylation by an ERK kinase [60,61,62,63]. AKT can phosphorylate BAD and BAX and regulate their proapoptotic function [64].

The role of IGF-1 has also been investigated in the setting of COVID-19. IGF-1 is an important factor for inflammation and immune regulation in the lung. Stimulation of IGF-1R in the process of lung inflammation activates the PI3K/AKT signaling pathway (Figure 1). Research has indicated the upregulation of IGF-1 and IGF-1R in the lung tissues of patients with ARDS related to COVID-19 [39]. Serum levels of IGF-1 may decline in more severe cases. It has been hypothesized that the blockage of IGF-1R may mitigate lung injury and decrease the risk of death in patients with COVID-19-related adult respiratory distress syndrome (ARDS) [39]. It has been shown that infection of lung epithelial cells with respiratory syncytial virus induces EGFR activation, which leads to increased inflammation in SARS-CoV infection [65], [66]. A number of viruses have been shown to activate p38 MAPK, including the herpes simplex virus [66]. Some of these viruses (for example, herpes simplex virus and measles) interfere with interferon signaling by inhibiting STAT phosphorylation [67,68].

## 5. Conclusions

The contribution of IGF-1 and IGF-1R to the mechanism of infection has been well documented in several virological studies, including studies on pneumonic viruses.

In general, viruses obtain greater benefits in activating rather than suppressing the PI3K/AKT signaling pathway. Viral activation of the PI3K/AKT signaling pathway slows the apoptosis rate and prolongs the period for viral replication.

Some viruses, such as ALV, ASFV, EV71, ZEBOV or HCV, utilize the PI3K/AKT signaling pathway during host cell entry. The upregulation of IGF-1 and IGF-1R has been observed in the lung tissues of patients with ARDS related to COVID-19. IGF-1R can also activate IRS protein and Shc adaptor protein, stimulate protein kinase phosphorylation, activate several proteins and stimulate cell proliferation. Moreover, to induce their release from cells, certain viruses leverage host transcription factors in the Ras/Raf/MEK/ERK pathway. IGF1/IGF-1R signaling also promotes cell differentiation and proliferation via the Ras/MAPK pathway, which is involved in oncogenesis processes in, for example, bovine leukemia virus infection. Some pneumonia viruses, such as syncytial virus, influenza, parainfluenza and calicivirus, can also use IGF-1R as a receptor. In a viral infection, abnormal expression of IGBPs has been observed. Viral infection can induce specific miRNAs, as revealed in studies of IGF-1 expression. Moreover, it has been shown that some viruses encode peptides similar to insulin or IGF-1 and thus affect host pathophysiology.

The IGF axis has become an attractive therapeutic target. Further studies regarding the linkage between viral infection and cellular factors involved in IGF-1 expression may provide new insights into the association between the aforementioned factors and receptors in other viral infections [36]. This research can help create new diagnostics and therapeutic tools to help in the treatment of many viral diseases, including COVID-19.

## Data Availability

Not Applicable.

## References

[1] Baxter R.C. (2014). IGF binding proteins in cancer: Mechanistic and clinical insights. Nat. Rev. Cancer.

[2] Gusscott S., Tamiro F., Giambra V., Weng A.P. (2019). Insulin-like growth factor (IGF) signaling in T-cell acute lymphoblastic leukemia. Adv. Biol. Regul..

[3] Morgan E.L., Macdonald A. (2020). Manipulation of JAK/STAT Signalling by High-Risk HPVs: Potential Therapeutic Targets for HPV-Associated Malignancies. Viruses.

[4] Ji W.-T., Liu H. (2008). PI3K-Akt Signaling and Viral Infection. Recent Pat. Biotechnol..

[5] Ardito F., Giuliani M., Perrone D., Troiano G., Lo Muzio L. (2017). The crucial role of protein phosphorylation in cell signaling and its use as targeted therapy (Review). Int. J. Mol. Med..

[6] DuShane J.K., Wilczek M.P., Mayberry C.L., Maginnis M.S. (2018). ERK Is a Critical Regulator of JC Polyomavirus Infection. J. Virol..

[7] Shi W., Hou X., Peng H., Zhang L., Li Y., Gu Z., Jiang Q., Shi M., Ji Y., Jiang J. (2014). MEK/ERK signaling pathway is required for enterovirus 71 replication in immature dendritic cells. Virol. J..

[8] Koczorowska M.M., Kwasniewska A., Gozdzicka-Jozefiak A. (2011). IGF1 mRNA isoform expression in the cervix of HPV-positive women with pre-cancerous and cancer lesions. Exp. Ther. Med..

[9] Merle P., Trepo C. (2009). Molecular Mechanisms Underlying Hepatocellular Carcinoma. Viruses.

[10] Romanelli R.J., LeBeau A.P., Fulmer C.G., Lazzarino D.A., Hochberg A., Wood T.L. (2007). Insulin-like growth factor type-I receptor internalization and recycling mediate the sustained phosphorylation of Akt. J. Biol. Chem..

[11] Sehat B., Tofigh A., Lin Y., Trocmé E., Liljedahl U., Lagergren J., Larsson O. (2010). SUMOylation mediates the nuclear translocation and signaling of the IGF-1 receptor. Sci. Signal..

[12] Solomon-Zemler R., Sarfstein R., Werner H. (2017). Nuclear insulin-like growth factor-1 receptor (IGF1R) displays proliferative and regulatory activities in non-malignant cells. PLoS ONE.

[13] Aleksic T., Chitnis M.M., Perestenko O.V., Gao S., Thomas P.H., Turner G.D., Protheroe A.S., Howarth M., Macaulay V.M. (2010). Type 1 insulin-like growth factor receptor translocates to the nucleus of human tumor cells. Cancer Res..

[14] Warsito D., Sjöström S., Andersson S., Larsson O., Sehat B. (2012). Nuclear IGF1R is a transcriptional co-activator of LEF1/TCF. EMBO Rep..

[15] Arainga M., Takeda E., Aida Y. (2012). Identification of bovine leukemia virus tax function associated with host cell transcription, signaling, stress response and immune response pathway by microarray-based gene expression analysis. BMC Genom..

[16] Kasprzak A., Kwasniewski W., Adamek A., Gozdzicka-Jozefiak A. (2017). Insulin-like growth factor (IGF) axis in cancerogenesis. Mutat. Res. Mutat. Res..

[17] Dale O.T., Aleksic T., Shah K.A., Han C., Mehanna H., Rapozo D.C.M., Sheard J.D.H., Goodyear P., Upile N.S., Robinson M. (2015). IGF-1R expression is associated with HPV-negative status and adverse survival in head and neck squamous cell cancer. Carcinogenesis.

[18] Yahya M.A., Sharon S.M., Hantisteanu S., Hallak M., Bruchim I. (2018). The role of the insulin-like growth factor 1 pathway in immune tumor microenvironment and its clinical ramifications in gynecologic malignancies. Front. Endocrinol..

[19] Nahor I., Abramovitch S., Engeland K., Werner H. (2005). The p53-family members p63 and p73 inhibit insulin-like growth factor-I receptor gene expression in colon cancer cells. Growth Horm. IGF Res..

[20] Abramovitch S., Glaser T., Ouchi T., Werner H. (2003). BRCA1-Sp1 interactions in transcriptional regulation of the IGF-IR gene. FEBS Lett..

[21] Jozefiak A., Pacholska-Bogalska J., Myga-Nowak M., Kedzia W., Kwasniewska A., Luczak M., Kedzia H., Gozdzicka-Jozefiak A. (2008). Serum and tissue levels of insulin-like growth factor-I in women with dysplasia and HPV-positive cervical cancer. Mol. Med. Rep..

[22] Li C., Harada A., Oh Y. (2012). IGFBP-3 sensitizes antiestrogen-resistant breast cancer cells through interaction with GRP78. Cancer Lett..

[23] Lodhia K.A., Tienchaiananda P., Haluska P. (2015). Understanding the Key to Targeting the IGF Axis in Cancer: A Biomarker Assessment. Front. Oncol..

[24] Liu B., Lee H.Y., Weinzimer S.A., Powell D.R., Clifford J.L., Kurie J.M., Cohen P. (2000). Direct functional interactions between insulin-like growth factor-binding protein-3 and retinoid X receptor-α regulate transcriptional signaling and apoptosis. J. Biol. Chem..

[25] Grkovic S., O’Reilly V.C., Han S., Hong M., Baxter R.C., Firth S.M. (2013). IGFBP-3 binds GRP78, stimulates autophagy and promotes the survival of breast cancer cells exposed to adverse microenvironments. Oncogene.

[26] Matilainen M., Malinen M., Saavalainen K., Carlberg C. (2005). Regulation of multiple insulin-like growth factor binding protein genes by 1α,25-dihydroxyvitamin D3. Nucleic Acids Res..

[27] Itoh M., Ide S., Takashima S., Kudo S., Nomura Y., Segawa M., Kubota T., Mori H., Tanaka S., Horie H. (2007). Methyl CpG-Binding Protein 2 (a Mutation of Which Causes Rett Syndrome) Directly Regulates Insulin-Like Growth Factor Binding Protein 3 in Mouse and Human Brains. J. Neuropathol. Exp. Neurol..

[28] Azar W.J., Zivkovic S., Werther G.A., Russo V.C. (2014). IGFBP-2 nuclear translocation is mediated by a functional NLS sequence and is essential for its pro-tumorigenic actions in cancer cells. Oncogene.

[29] Wang G.K., Hu L., Fuller G.N., Zhang W. (2006). An interaction between insulin-like growth factor-binding protein 2 (IGFBP2) and integrin α5 is essential for IGFBP2-induced cell mobility. J. Biol. Chem..

[30] Fu P., Yang Z., Bach L.A. (2013). Prohibitin-2 binding modulates insulin-like growth factor-binding protein-6 (IGFBP-6)-induced rhabdomyosarcoma cell migration. J. Biol. Chem..

[31] Weigel K.J., Jakimenko A., Conti B.A., Chapman S.E., Kaliney W.J., Leevy W.M., Champion M.M., Schafer Z.T. (2014). CAF-Secreted IGFBPs Regulate Breast Cancer Cell Anoikis. Mol. Cancer Res..

[32] Diehl N., Schaal H. (2013). Make yourself at home: Viral hijacking of the PI3K/Akt signaling pathway. Viruses.

[33] Ehrhardt C., Wolff T., Pleschka S., Planz O., Beermann W., Bode J.G., Schmolke M., Ludwig S. (2007). Influenza A virus NS1 protein activates the PI3K/Akt pathway to mediate antiapoptotic signaling responses. J. Virol..

[34] Griffiths C.D., Bilawchuk L.M., McDonough J.E., Jamieson K.C., Elawar F., Cen Y., Duan W., Lin C., Song H., Casanova J.-L. (2020). IGF1R is an entry receptor for respiratory syncytial virus. Nature.

[35] Kumar D., Broor S., Rajala M.S. (2016). Interaction of Host Nucleolin with Influenza A Virus Nucleoprotein in the Early Phase of Infection Limits the Late Viral Gene Expression. PLoS ONE.

[36] Honda T., Fujino K., Okuzaki D., Ohtaki N., Matsumoto Y., Horie M., Daito T., Itoh M., Tomonaga K. (2011). Upregulation of insulin-like growth factor binding protein 3 in astrocytes of transgenic mice that express Borna disease virus phosphoprotein. J. Virol..

[37] Elawar F., Griffiths C.D., Zhu D., Bilawchuk L.M., Jensen L.D., Forss L., Tang J., Hazes B., Drews S.J., Marchant D.J. (2017). A Virological and Phylogenetic Analysis of the Emergence of New Clades of Respiratory Syncytial Virus. Sci. Rep..

[38] Iwakiri D., Eizuru Y., Tokunaga M., Takada K. (2003). Autocrine Growth of Epstein-Barr Virus-Positive Gastric Carcinoma Cells Mediated by an Epstein-Barr Virus-Encoded Small RNA. Cancer Res..

[39] Winn B.J. (2020). Is there a role for insulin-like growth factor inhibition in the treatment of COVID-19-related adult respiratory distress syndrome?. Med. Hypotheses.

[40] Altindis E., Cai W., Sakaguchi M., Zhang F., GuoXiao W., Liu F., De Meyts P., Gelfanov V., Pan H., DiMarchi R. (2018). Viral insulin-like peptides activate human insulin and IGF-1 receptor signaling: A paradigm shift for host-microbe interactions. Proc. Natl. Acad. Sci. USA.

[41] Carotti S., Guarino M.P.L., Valentini F., Porzio S., Vespasiani-Gentilucci U., Perrone G., Zingariello M., Gallo P., Cicala M., Picardi A. (2018). Impairment of GH/IGF-1 Axis in the Liver of Patients with HCV-Related Chronic Hepatitis. Horm. Metab. Res..

[42] Wang J., Li Y.-C., Deng M., Jiang H.-Y., Guo L.-H., Zhou W.-J., Ruan B. (2017). Serum insulin-like growth factor-1 and its binding protein 3 as prognostic factors for the incidence, progression, and outcome of hepatocellular carcinoma: A systematic review and meta-analysis. Oncotarget.

[43] Scharf J.G., Ramadori G., Dombrowski F. (2000). Analysis of the IGF axis in preneoplastic hepatic foci and hepatocellular neoplasms developing after low-number pancreatic islet transplantation into the livers of streptozotocin diabetic rats. Lab. Investig..

[44] Yang X.-J. (2005). Multisite protein modification and intramolecular signaling. Oncogene.

[45] Crow M.S., Lum K.K., Sheng X., Song B., Cristea I.M. (2016). Diverse mechanisms evolved by DNA viruses to inhibit early host defenses. Crit. Rev. Biochem. Mol. Biol..

[46] Wojcechowskyj J.A., Didigu C.A., Lee J.Y., Parrish N.F., Sinha R., Hahn B.H., Bushman F.D., Jensen S.T., Seeholzer S.H., Doms R.W. (2013). Quantitative phosphoproteomics reveals extensive cellular reprogramming during HIV-1 entry. Cell Host Microbe.

[47] Jakubiec A., Jupin I. (2007). Regulation of positive-strand RNA virus replication: The emerging role of phosphorylation. Virus Res..

[48] Keating J.A., Striker R. (2012). Phosphorylation events during viral infections provide potential therapeutic targets. Rev. Med. Virol..

[49] Yu Y., Alwine J.C. (2002). Human Cytomegalovirus Major Immediate-Early Proteins and Simian Virus 40 Large T Antigen Can Inhibit Apoptosis through Activation of the Phosphatidylinositide 3′-OH Kinase Pathway and the Cellular Kinase Akt. J. Virol..

[50] Datta S.R., Brunet A., Greenberg M.E. (1999). Cellular survival: A play in three akts. Genes Dev..

[51] Zhang L., Wu J., Ling M.T., Zhao L., Zhao K.-N. (2015). The role of the PI3K/Akt/mTOR signalling pathway in human cancers induced by infection with human papillomaviruses. Mol. Cancer.

[52] Ganti K., Broniarczyk J., Manoubi W., Massimi P., Mittal S., Pim D., Szalmas A., Thatte J., Thomas M., Tomaić V. (2015). The Human Papillomavirus E6 PDZ Binding Motif: From Life Cycle to Malignancy. Viruses.

[53] Accardi R., Rubino R., Scalise M., Gheit T., Shahzad N., Thomas M., Banks L., Indiveri C., Sylla B.S., Cardone R.A. (2011). E6 and E7 from Human Papillomavirus Type 16 Cooperate To Target the PDZ Protein Na/H Exchange Regulatory Factor 1. J. Virol..

[54] Wu H.H., Wu J.Y., Cheng Y.W., Chen C.Y., Lee M.C., Goan Y.G., Lee H. (2010). cIAP2 upregulated by E6 oncoprotein via epidermal growth factor receptor/phosphatidylinositol 3-kinase/AKT pathway confers resistance to cisplatin in human papillomavirus 16/18-infected lung cancer. Clin. Cancer Res..

[55] Wu J., Chen J., Zhang L., Zhao P.P.M., Zhao K.-N. (2014). Four Major Factors Regulate Phosphatidylinositol 3-kinase Signaling Pathway in Cancers Induced by Infection of Human Papillomaviruses. Curr. Med. Chem..

[56] Charette S.T., McCance D.J. (2007). The E7 protein from human papillomavirus type 16 enhances keratinocyte migration in an Akt-dependent manner. Oncogene.

[57] Liu R., Chen Y., Liu G., Li C., Song Y., Cao Z., Li W., Hu J., Lu C., Liu Y. (2020). PI3K/AKT pathway as a key link modulates the multidrug resistance of cancers. Cell Death Dis..

[58] Liefers-Visser J.A.L., Meijering R.A.M., Reyners A.K.L., van der Zee A.G.J., de Jong S. (2017). IGF system targeted therapy: Therapeutic opportunities for ovarian cancer. Cancer Treat. Rev..

[59] Zachos G., Koffa M., Preston C.M., Clements J.B., Conner J. (2001). Herpes simplex virus type 1 blocks the apoptotic host cell defense mechanisms that target Bcl-2 and manipulates activation of p38 mitogen-activated protein kinase to improve viral replication. J. Virol..

[60] Pétigny-Lechartier C., Duboc C., Jebahi A., Louis M.-H., Abeilard E., Denoyelle C., Gauduchon P., Poulain L., Villedieu M. (2017). The mTORC1/2 Inhibitor AZD8055 Strengthens the Efficiency of the MEK Inhibitor Trametinib to Reduce the Mcl-1/[Bim and Puma] ratio and to Sensitize Ovarian Carcinoma Cells to ABT-737. Mol. Cancer Ther..

[61] Hata A.N., Engelman J.A., Faber A.C. (2015). The BCL2 Family: Key Mediators of the Apoptotic Response to Targeted Anticancer Therapeutics. Cancer Discov..

[62] O’Reilly L.A., Kruse E.A., Puthalakath H., Kelly P.N., Kaufmann T., Huang D.C.S., Strasser A. (2009). MEK/ERK-mediated phosphorylation of Bim is required to ensure survival of T and B lymphocytes during mitogenic stimulation. J. Immunol..

[63] Moustafa-Kamal M., Gamache I., Lu Y., Li S., Teodoro J.G. (2013). BimEL is phosphorylated at mitosis by Aurora A and targeted for degradation by βTrCP1. Cell Death Differ..

[64] Deng J. (2017). How to unleash mitochondrial apoptotic blockades to kill cancers?. Acta Pharm. Sin. B.

[65] Monick M.M., Cameron K., Staber J., Powers L.S., Yarovinsky T.O., Koland J.G., Hunninghake G.W. (2005). Activation of the Epidermal Growth Factor Receptor by Respiratory Syncytial Virus Results in Increased Inflammation and Delayed Apoptosis. J. Biol. Chem..

[66] Hemmat N., Asadzadeh Z., Ahangar N.K., Alemohammad H., Najafzadeh B., Derakhshani A., Baghbanzadeh A., Baghi H.B., Javadrashid D., Najafi S. (2021). The roles of signaling pathways in SARS-CoV-2 infection; lessons learned from SARS-CoV and MERS-CoV. Arch. Virol..

[67] Takeuchi K., Kadota S., Takeda M., Miyajima N., Nagata K. (2003). Measles virus V protein blocks interferon (IFN)-α/β but not IFN-γ signaling by inhibiting STAT1 and STAT2 phosphorylation. FEBS Lett..

[68] Bordignon V., Di Domenico E., Trento E., D’Agosto G., Cavallo I., Pontone M., Pimpinelli F., Mariani L., Ensoli F. (2017). How Human Papillomavirus Replication and Immune Evasion Strategies Take Advantage of the Host DNA Damage Repair Machinery. Viruses.

